# Treatment With EkoSonic™ Endovascular System (EKOS®) of Massive Pulmonary Thrombosis Following Recovery From COVID-19 Infection

**DOI:** 10.7759/cureus.30467

**Published:** 2022-10-19

**Authors:** Ugur Cetingok, Cayan Akkoyun, Zulkuf Isik, Orhan Gungor

**Affiliations:** 1 Department of Cardiovascular Surgery, Alanya Anadolu Hospital, Antalya, TUR; 2 Department of Cardiology, Uzmanlar Hospital, Yalova, TUR; 3 Department of Pulmonology, Atakent Hospital, Yalova, TUR; 4 Department of Cardiovascular Surgery, Cesme Public Hospital, Izmir, TUR

**Keywords:** systemic thrombolysis, ekos catheter, accelerated thrombolysis, pulmonary embolism (pe), pulmonary artery thrombosis, covid-19 infection

## Abstract

COVID-19 infection caused by the new coronavirus called severe acute respiratory syndrome coronavirus 2 (SARS-CoV-2) is an infection with symptoms and results ranging from mild flu-like symptoms to severe respiratory failure leading to death. The risk of thrombosis increases due to hypercoagulation in COVID-19 infection. All causes (endothelial injury, stasis, and hypercoagulopathy) known as Virchow's triad contribute to thrombosis in COVID-19 infection. However, the pathogenesis of hypercoagulability in COVID-19 is still unknown. In this article, we discuss the unique multiple thrombosis events following recovery from COVID-19 infection and our treatment strategy for pulmonary thrombosis. The patient had symptoms of dry cough, fever, and myalgia two months ago. His polymerase chain reaction (PCR) test for COVID-19 was positive, but there was no need for hospitalization. His symptoms resolved within seven days. But, thrombosis of the superior mesenteric artery (SMA) occurred one month after the COVID-19 infection, and bowel resection was performed. He was admitted to our hospital with dyspnea, chest pain, palpitations, and hoarseness. Further evaluation showed tachycardia, hypotension, tachypnea, and anxiety. Peripheral oxygen saturation (SpO_2_) was 86% at room air. He had hemodynamic instability, right ventricular (RV) dysfunction, and D-Dimer elevation. Pulmonary Embolism Severity Index (PESI) was calculated as 149. The patient was in the high-risk group. Our Pulmonary Embolism Response Team (PERT) decided to apply catheter-directed thrombolysis (CDT) for the treatment of pulmonary thrombosis. The EkoSonic™ Endovascular System (EKOS) (Boston Scientific Corporation, Marlborough, USA) was used for the treatment.

## Introduction

The disease, which is caused by the SARS-CoV-2 virus and called COVID-19 infection, at the end of 2019 rapidly spread around the world, causing the death of millions of people. The pandemic of COVID-19 still continues. However, vaccination applications have reduced the need for intensive care and deaths due to COVID-19 infection. Thrombosis incidence is high (31%) in patients requiring intensive care [1]. This situation makes the treatment of the disease even more difficult.

Options for the current treatment of pulmonary embolism (PE); anticoagulation, fractionated heparin infusion or low molecular weight heparin (LMWH), thrombolytics, and catheter-directed thrombolysis (CDT). The choice of treatment depends on the patient's condition.

The EkoSonic™ Endovascular System (EKOS) (Boston Scientific Corporation, Marlborough, USA) provides an ultrasound-facilitated, controlled and selective infusion of physician-specified fluids, including thrombolytics into the vasculature. U.S. Food and Drug Administration (FDA) approved EKOS for the treatment of peripheral vascular thrombosis and embolism (arterial and venous) in 2004, and for the treatment of PE in 2014. It is also called ultrasound-assisted thrombolysis (USAT), ultrasound-accelerated thrombolysis, and ultrasound-enhanced thrombolysis.

All of the studies on the EKOS catheter showed rapid improvement and restoration in the right ventricular (RV) function. The treatment with the EKOS provides faster thrombolysis with fewer thrombolytic agents than standard catheter-directed thrombolysis (SCDT). However, Avgerinos et al. reported similar pulmonary artery thrombus reduction in SCDT and USAT when comparing mean lytic doses and lysis times with the SUNSET sPE trial [2].

## Case presentation

A 49-year-old male patient was brought to the emergency department in July 2021 with complaints of palpitation, dyspnea, chest pain, anxiety, hypotension, hoarseness and coldness of the left hand. We learned from the patient's history he had fever, cough and myalgia symptoms and a positive polymerase chain reaction (PCR) test for COVID-19 two months ago and was treated as an outpatient. He recovered within a week. But thrombosis of the superior mesenteric artery (SMA) occurred one month after the COVID-19 infection and bowel resection was performed.

During his evaluation, sinus tachycardia (144 beats/min), hypotension (80/55 mmHg), and tachypnea (30/min) were detected. Oxygen saturation (SpO_2_) was 86% room air. The pulses of the left upper extremity were negative due to occlusion in the left subclavian artery. However, there was mild ischemic finding. Therefore, an emergency intervention was not considered for the left subclavian artery occlusion.

The patient was admitted to the intensive care unit (ICU). All vital signs were monitored. The treatment for hypotension, hypoxia, and chest pain was started. Enlarged RV (RV/left ventricular (LV) ratio: 1.1), moderate tricuspid valve insufficiency and high pulmonary artery pressure (50 mmHg) were detected by the bedside echocardiography. The LV systolic functions were normal. The venous color doppler ultrasound for the deep veins of the legs was performed. There was no sign of thrombosis.

The laboratory tests showed an increased sedimentation rate (70 mm/h) and a high level of D-dimer (9547 ng/dl). Hypoxia, hypercapnia (partial pressure of oxygen - pO_2_ 75 mmHg, partial pressure of carbon dioxide - pCO_2_ 47 mmHg) and acidosis (pH 7.29 ) were detected in the arterial blood gas analysis (Table 1 ). The SARS-CoV-2 test was negative.

**Table 1:** Laboratory test results of the patient

Name of test	1^st^ day	2^nd^ day	3^rd^ day	Reference Range
Hemoglobin (g/dl)	10.3	10.7	10.1	13.5-17.5
White blood cell (10³/µL)	6.33	5.37	5.89	4-11
Platelet (10³/µL)	319	323	313	142-424
Prothrombine time (sec)	12.1	18.1	18.3	10.5-15.5
Activated parsiyel thromboplastin time (sec)	20.8	22	19.4	23-39
D-Dimer (ng/dl)	9547	3670	1164	0-500
Troponin I (ng/ml)	0.01	0.02	0.01	0-0.16
Pro-Brain natriuretic peptide (pg/dl)	11.7	26.3	20.6	0-300
Blood urea nitrogen (mmol/L)	16	12	15	10-50
Creatinine (µmol/L)	0.74	0.62	0.68	0.6-1.3
Sodium (mEq/L)	137	136	135	133-146
Potasium (mEq/L)	4.3	3.7	3.8	3.5-5.1
Alanine aminotransferase (U/L)	23	18	15	5-50
Aspartate aminotransferase (U/L)	21	19	18	5-50
Lactate dehydrogenase (U/L)	205	304	340	135-214
Sedimentation rate (mm/hour)	70	60	37	0-15
C-reactive protein (mg/dl)	25	15	5	0-5
Arterial pO_2_ (mmHg)	75.6	97.4	97.8	80-100
Arterial pCO_2_ (mmHg)	47.1	34.4	27.2	35-45
Arterial pH	7.29	7.47	7.48	7.35-7.45
SARS-CoV-2 test	Negative			

The Pulmonary Embolism Severity Index (PESI) score was calculated to be 149. The patient was a Class V and in the very high mortality risk group according to the PESI (Table 2 ).

**Table 2:** Calculated Pulmonary Embolism Severity Index (PESI)

Parameter	Original PESI score
Age	+ 49
Sex (male)	+ 10
History of cancer	No
History of heart failure	No
History of chronic lung disease	No
Heart rate ≥ 110 beats / min	Yes + 20
Systolic blood pressure < 100 mmHg	Yes + 30
Respiratory rate ≥ 30 breaths / min	Yes + 20
Temperature < 36 °C	No
Altered mental status (Disorientation, lethargy, stupor, or coma)	No
O_2_ saturation < 90%	Yes + 20
Class V: >125, very high mortality risk (10.0-24.5%)	Total 149

**Figure 1:**
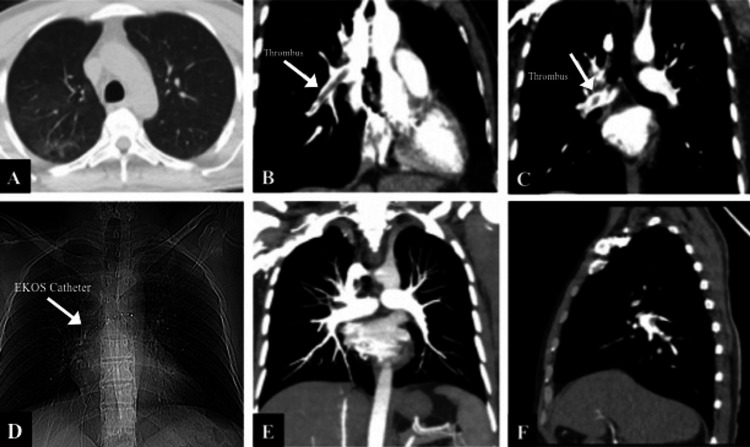
The CT findings before the EKOS treatment (A, B, C ) and at the 12th hour of the EKOS treatment (D, E, F) A: Ground-glass opacities in the right lobe; B: Massive thrombus in the right pulmonary artery; C: Filling defects due to thrombus in the segmental branches; D: View of the EKOS catheter; E: Totally cleared the right pulmonary artery and anterior truncus; F: Thrombus fragments in the posterior ascending artery and the common basal arteries EKOS: The EkoSonic™ Endovascular System (EKOS) (Boston Scientific Corporation, Marlborough, USA)

A CT scan revealed ground-glass opacities in the right upper lobe related to the previous COVID-19 pneumonia. It involved less than 10% of the lung (Figure 1A). However, CT pulmonary angiography (CTPA) showed a massive thrombus in the right pulmonary artery (Figure 1B) and filling defects in its segmental branches (Figure 1C).

**Figure 2:**
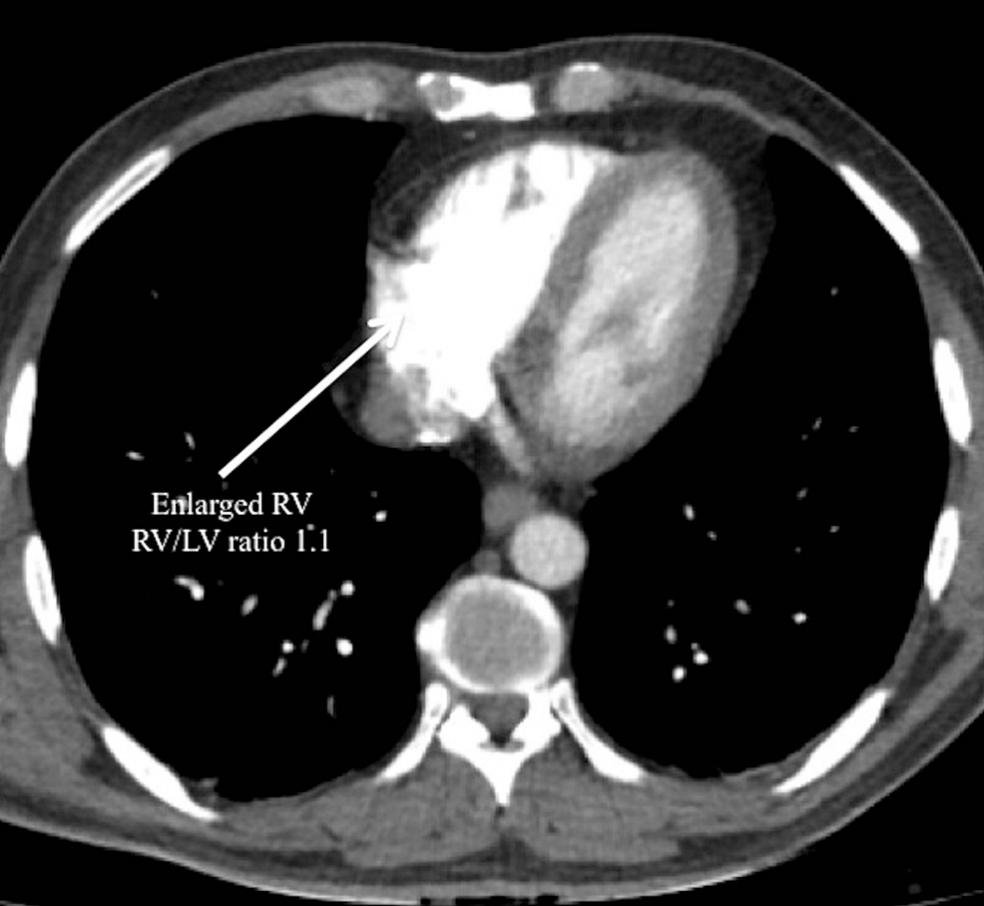
RV enlargement (RV/LV ratio 1.1) RV: right ventricle; LV: left ventricle

The RV enlargement (RV/LV ratio 1.1) was seen on the CT (Figure 2).

**Figure 3:**
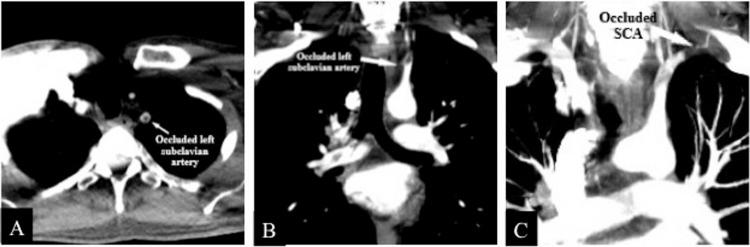
CTA findings of the left SCA occlusion. A: Filling defect in the left SCA, B: Occlusion of the left SCA, C: Partial thrombus before the left vertebral artery in the left SCA, total occlusion after the vertebral artery, patent the left vertebral artery. SCA: subclavian artery

The level of arterial occlusion causing left upper extremity ischemia was determined on the CT angiography (CTA). The proximal segment of the left subclavian artery (SCA) was occluded (Figure 3).

**Figure 4:**
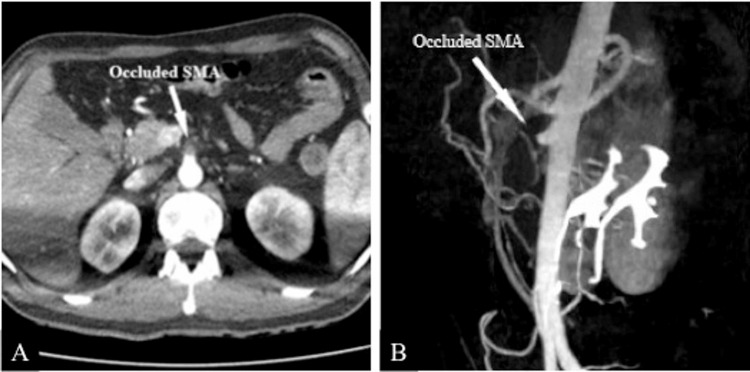
CTA findings of the SMA occlusion. A: Axial view; total occlusion of the SMA; B: Sagittal view; total occlusion of the SMA, no recanalization. SMA: superior mesenteric artery

The CTA also detected thrombosis of the superior mesenteric artery (SMA) resulting in bowel resection. Recanalization was not observed in the SMA (Figure 4).

All examinations and consultations were completed within one hour.

Our Pulmonary Embolism Response Team (PERT) consisting of pulmonology, cardiology, cardiovascular surgery and anesthesiology specialists discussed the treatment options for the patient. The patient had undergone a major surgical operation such as a bowel resection 28 days ago. He had hemodynamic instability, right ventricular dysfunction, and D-Dimer elevation. PESI was calculated as 149, and he was in the high-risk group. The treatment with USAT was decided. Because this method allows treatment with a lower dose of thrombolytic agent compared to systemic treatment and the risk of bleeding is significantly reduced. EKOS was used for the treatment.

**Figure 5:**
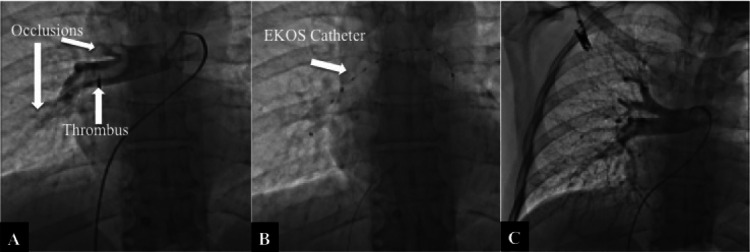
A: Pulmonary angiography before the EKOS treatment; B: EKOS catheter was advanced across the treatment site; C: Pulmonary angiography at the 18th hour of treatment - the thrombi were completely dissolved EKOS: The EkoSonic™ Endovascular System (EKOS) (Boston Scientific Corporation, Marlborough, USA)

The patient was transferred to the angiography laboratory. A 7F sheath was placed on the right common femoral vein. Bilateral pulmonary angiography was performed. The pulmonary angiography revealed 90% narrowing in the right pulmonary artery and totally occlusion in the anterior truncus, posterior ascending and common basal arteries caused by a thrombus (Figure 5A). The EKOS catheter (135 x 12 cm) was placed in the right pulmonary artery with the guidance of a 0.035" stiff hydrophilic guide-wire. The guide-wire was exchanged with the ultrasound core (Figure 5B). The catheter was primed with 2 ml of alteplase (rt-PA) before connecting to the control unit. 5 mg alteplase (rt-PA) was given into the thrombus on-table bolus. Selective thrombolysis was started with rt-PA 1 mg per hour. The catheter was fixed and the patient was transferred to the ICU.

Symptoms regressed rapidly within an hour. The arterial blood gas values improved (pO_2_ 97 mmHg, pCO_2_ 34 mmHg, pH 7.47) and SpO_2_ was measured at 99%.

Control CTPA was performed at the 12th hour of the EKOS treatment. The right pulmonary artery and anterior truncus were totally cleared (Figure 1E). Thrombus fragments in the posterior ascending artery and the common basal arteries were observed (Figure 1F). It was decided to continue the treatment with the same dose of rt-PA for another 6 hours.

Control pulmonary angiography was performed at the 18th hour of the EKOS treatment. The thrombi in the pulmonary artery and its branches had completely dissolved (Figure 5C).

The patient was administered 25 mg of rt-PA during the treatment. The treatment was continued with 60 mg of enoxaprin sodium (Clexane 60 mg / 0.6 mL / 6000 anti-Xa IU) twice a day. Coumarin was added to therapy. No complications were observed during the treatment. The patient was discharged on the seventh postoperative day, with the dose of coumarin adjusted to be between 2.5 and 3.0 with an international normalized ratio (INR).

At the 1st, 3rd and 6th-month follow-ups, the patient did not have any symptoms, and the physical examination, echocardiography and chest X-ray were normal.

## Discussion

COVID-19 infection covers a wide spectrum, from mild flu-like symptoms to severe respiratory failure, shock and/or multi-organ dysfunction leading to death [3]. PE or thrombosis is more common in patients who are in the ICU for COVID-19 disease. Poissy et al. performed CTPA for the diagnosis of PE in 34 (31.8%) of 107 COVID-19 patients hospitalized in the ICU within a 1-month period between February and March 2020. PE was defined in 22 (20.6%) of the patients. Deep vein thrombosis (DVT) was detected in only three (13.6%) of the patients diagnosed with PE [4]. However, it may occur due to increased thrombogenicity in the outpatients and healed patients. The rates of PE developing after discharge from the hospital are low and reported as 2% in the first 6 weeks [5].

The disposition to thrombosis in COVID-19 infection is due to hypercoagulation, immunothrombosis, and endothelial injury [6]. The increased tendency to thrombosis is called COVID-19-associated coagulopathy [7]. The pathogenesis of hypercoagulability is still unknown. Cell damage due to virus invasion into the endothelial cells, stasis due to immobility, elevated factor VIII and fibrinogen, circulating prothrombotic microparticles, neutrophil extracellular traps and increased viscosity are possible causes for hypercoagulation in COVID-19 infection [8-9]. Elevated of D-dimer levels correlate with the severity of the disease [10]. However, there is not enough evidence to recommend the use of biomarkers in diagnostic evaluation. PE should be considered if a patient with COVID-19 disease has hemodynamic instability or poor gas exchange that is unexplained or disproportionate to the course of the disease [11].

Identification of risk in the patient helps in the choice of the therapy in PE. European Society of Cardiology (ESC) classifies acute PE as high risk, intermediate risk, and low risk. PESI is a very useful scoring method for risk stratification in patients with PE. PERT assessments define the hemodynamic and cardiopulmonary status, provide risk stratification and define the best treatment modality for the patients [11].

The National PERT consortium emphasized that the indications and contraindications for thrombolysis have not changed in COVID-19 patients complicated with PE, systemic thrombolysis is an alternative if the invasive approach cannot be used, that the risk-benefit ratio of medical and interventional treatment may require adjustment, and the PERT evaluation provides an opportunity to evaluate complex interventional options [11].

The standard treatment for PE is intravenous heparin or LMWH and followed by oral anticoagulants. The massive PE and submassive PE that causes RV failure can be treated with systemic thrombolytic treatment (STT). Although STT is most beneficial when the treatment is started within 48 hours of the onset of the symptoms, thrombolysis may be beneficial in patients who have symptoms for 6-14 days [12]. However, STT can cause fatal bleeding. Major bleeding due to the STT is approximately 20% and intracranial hemorrhage (ICH) is 2-3% [13].

The surgical pulmonary thrombectomy is indicated in the massive PE and the high-risk submassive PE in those who cannot receive the STT or in those who have failed the STT, in those with right heart thrombus at risk for left-sided embolism [14]. The pulmonary embolectomy is performed reliably on the beating heart with cardiopulmonary bypass without aortic clamping in selected cases [15].

On the other hand, CDT is much safer than systemic therapy. The risk of major bleeding for the CDT is lower than the STT (CDT - 7%, STT - 20%,). It is considered an appropriate treatment alternative in high-risk PE patients [16]. There are several devices are available for the CDT. The most common of these are summarized in Table 3.

**Table 3:** Most common techniques and device samples for CDT CDT: catheter-directed thrombolysis

Technique	Device
Without thrombolysis	
Aspiration thrombectomy	Angiovac suction cannula (AngioDynamics, Latham, USA)
Mechanical thrombectomy	FlowTriever (Inari Medical, Irvine, USA)
Rheolytic thrombectomy	Angiojet PE (Boston Scientific Corp, Marlborough, USA)
Rotational thrombectomy	Aspirex Thrombectomy (Straub Medical AG, Wangs, Switzerland), Cleaner Thrombectomy (Argon Medical Device Inc, Plano, USA)
With thrombolysis	
Catheter-directed thrombolysis	Uni-Fuse (AngioDynamics, Latham, USA)
Ultrason-assisted catheter-directed thrombolysis	Ekosonic Endovascular System (EKOS) (Boston Scientific Corp, Marlborough, USA)
Pharmacomechanical thrombolysis	Angiojet PE / Power pulse (Boston Scientific Corp, Marlborough, USA)

EKOS is different from CDT, with high-frequency, low-energy ultrasound waves. The ultrasound waves accelerate the penetration of thrombolytic agents into the clot and make them break down faster with lower drug doses. EKOS provides faster thrombolysis with fewer thrombolytic agents than SCDT. Drug-related complications are minimized with EKOS treatment. According to one study, 84% of patients recovered within 2 hours of the start of treatment using one-fifth of the systemic dose [17]. However, the results of the SUNSET sPE trial (Standard vs. Ultrasound-Assisted Catheter Thrombolysis for Submassive Pulmonary Embolism) were published by Avgerinos et al. in June, 2021. From 2016 through 2020, a total of 82 patients randomly assigned to 1:1 to USAT or SCDT with similar demographic characteristics, comorbidity, risk factors, and PE severity were enrolled in this multicenter, randomized trial. Pulmonary arterial thrombus reduction was similar for the two groups when the mean lytic doses and lysis times were compared [2].

EKOS received FDA approval for the treatment of pulmonary embolism in 2014. All four completed studies (ULTIMA trial, SEATTLE II trial, PERFECT trial, and OPTALYSE trial) on the EKOS catheter demonstrated rapid improvement and restoration of RV function as a result of its treatment [18]. In addition, ICH was not reported in these four studies.

ESC and PERT Consortium recommendations for CDT in acute PE are shown in Table 4.

**Table 4:** ESC and PERT Consortium recommendations for CDT in acute PE ECMO, extracorporeal membrane oxygenation; ESC, European Society of Cardiology; LOE, level of evidence; PE, pulmonary embolism; PERT, Pulmonary Embolism Response Team; RV, right ventricle; STT, systemic thrombolysis, CDT, catheter-directed thrombolysis Source: [19,20]

Risk Level	ESC	PERT Consortium	
Intermediate- or Intermediate-high risk	Consider catheter-directed treatment in those patients with hemodynamic deterioration already receiving anticoagulation therapy. (Class IIa recommendation/LOE C)	Consider catheter-directed thrombolysis if: (1) The risk of clinical deterioration based on hemodynamics, degree of RV dysfunction, end-organ ischemia, gas exchange. (2) No absolute contraindications to STT.	
High-risk	a) Catheter therapies should be considered when STT is contraindicated or has failed. (Class IIa recommendation/LOE C) (b) Catheter therapies may be used in conjunction with ECMO in those with refractory circulatory collapse or cardiac arrest. (Class IIa recommendation/LOE C)	Consider catheter-directed thrombolysis if relative contraindications to STT.	

## Conclusions

The EKOS treatment was applied to the patient without any complications. With the treatment, SpO_2_, arterial pO_2_ and pCO_2_, arterial pH, arterial blood pressure, heart rate, and inspiratory rate improved within 1 hour. The pulmonary thrombus load decreased at 6 hours, and complete cleaning of the pulmonary arterial bed was achieved in 18 hours. Adequate treatment was provided with a low-dose thrombolytic agent using 25 mg alteplase (rt-PA) during the treatment.

Rapid evaluation of the patient by PERT and immediate treatment of PE according to its severity are very important in high-risk PE. Ultrasound accelerated thrombolysis (EkoSonic™ Endovascular System - EKOS) is a safe and effective treatment in COVID-19-related pulmonary thrombosis. USAT is becoming more and more commonly used over time in high-risk and intermediate-high-risk PEs. This treatment provides faster and safer thrombolysis with a lesser amount of thrombolytic agents, and the patient can be discharged in a shorter time.
